# New diagnostic criteria for metopic ridges and trigonocephaly: a 3D geometric approach

**DOI:** 10.1186/s13023-024-03197-8

**Published:** 2024-05-18

**Authors:** Kevin Bloch, Maya Geoffroy, Maxime Taverne, Lara van de Lande, Eimear O’Sullivan, Ce Liang, Giovanna Paternoster, Mehran Moazen, Sébastien Laporte, Roman Hossein Khonsari

**Affiliations:** 1 Service de chirurgie maxillofaciale et chirurgie plastique, Hôpital Necker - Enfants malades, Assistance Publique - Hôpitaux de Paris, CRMR CRANIOST, Faculté de Médecine, Université Paris Cité, Paris, France; 2 Institut de Biomécanique Humaine Georges Charpak, Arts et Métiers Institute of Technology, Paris, France; 3 Laboratoire ‘Forme et Croissance du Crâne’, Hôpital Necker - Enfants malades, Assistance Publique - Hôpitaux de Paris, Paris, France; 4 Craniofacial Unit, Great Ormond Street Hospital for Children; UCL Great Ormond Street Institute of Child Health, London, UK; 5 Department of Oral and Maxillofacial Surgery, Erasmus Medical Centre, Rotterdam, the Netherlands; 6 Department of Computing, Imperial College London, London, UK; 7 Department of Mechanical Engineering, University College London, London, UK; 8 Service de Neurochirurgie, Hôpital Necker - Enfants malades, Assistance Publique - Hôpitaux de Paris, CRMR CRANIOST, Paris, France

**Keywords:** Craniosynostosis, Morphometry, Diagnostic tool, Geometric morphometrics, Artificial intelligence

## Abstract

**Background:**

Trigonocephaly occurs due to the premature fusion of the metopic suture, leading to a triangular forehead and hypotelorism. This condition often requires surgical correction for morphological and functional indications. Metopic ridges also originate from premature metopic closure but are only associated with mid-frontal bulging; their surgical correction is rarely required. Differential diagnosis between these two conditions can be challenging, especially in minor trigonocephaly.

**Methods:**

Two hundred seven scans of patients with trigonocephaly (90), metopic rigdes (27), and controls (90) were collected. Geometric morphometrics were used to quantify skull and orbital morphology as well as the interfrontal angle and the cephalic index. An innovative method was developed to automatically compute the frontal curvature along the metopic suture. Different machine-learning algorithms were tested to assess the predictive power of morphological data in terms of classification.

**Results:**

We showed that control patients, trigonocephaly and metopic rigdes have distinctive skull and orbital shapes. The 3D frontal curvature enabled a clear discrimination between groups (sensitivity and specificity > 92%). Furthermore, we reached an accuracy of 100% in group discrimination when combining 6 univariate measures.

**Conclusion:**

Two diagnostic tools were proposed and demonstrated to be successful in assisting differential diagnosis for patients with trigonocephaly or metopic ridges. Further clinical assessments are required to validate the practical clinical relevance of these tools.

**Supplementary Information:**

The online version contains supplementary material available at 10.1186/s13023-024-03197-8.

## Background

Premature prenatal metopic suture fusion constraints frontal cranial growth and causes trigonocephaly (TG) [1], characterized by triangular forehead, biparietal widening, and hypotelorism. Metopic ridges (MR) correspond to metopic suture ossification, responsible for an isolated clinically palpable midline forehead ridge.

The diagnosis of TG is straightforward in severe forms, but differentiating moderate forms from MR can be challenging. Clinical and radiological signs and anthropometric measurements have been proposed for differential diagnosis [2, 3], such as the frontal 3D curvature [4]. Here we assessed 3D cranial shape in TG, MR, and controls using geometric morphometrics and introduced anthropometric measures that can discriminate these conditions.

## Material and methods

### Study population

All patients with non-syndromic TG that benefited from fronto-orbital advancement at Necker—Enfants Malades Hospital (Paris), at the National Reference Center for Craniofacial Malformations from 2004 until 2019 with an available digital pre-operative CT-scan were included using a local data warehouse [5]. All patients diagnosed with MR managed in the same center during the same period with available digital CT-scans were included. These patients had benefited from CT-scans before referral to our center as local pediatric teams had suspected TG; they thus represented MR cases that had initially raised diagnostic issues. Control age-matched patients were included with CT-scans performed for acute headache, soft-tissue infections, epilepsy, or trauma. All control CT-scans were assessed by two independent reviewers (surgeon and radiologist) to exclude skull fractures and malformations and ensure sufficient quality for 3D reconstruction. Age and gender were recorded for all patients. The study was approved by the local ethical committee and patients were informed of the conduct of the investigations.

### Registration and skull shape quantification

CT-scans were segmented using 3D Slicer [6] and exported as STL files. The intracranial cavity was segmented for intracranial volume (ICV) computation. Skull surfaces were rigidly aligned with a template corresponding to a mean normal external vault surface [7] using 8 landmarks (Supp. Mat. 1A and 1B). A non-rigid iterative closest point algorithm (NICP) was then used to deform the template mesh and establish a dense correspondence with each of the input meshes, resulting in subject-specific surface meshes with the same topology as the template (Supp. Mat. 2).

### Orbital shape quantification

Orbital shapes were characterized by placing 8 anatomical landmarks and 50 semi-landmarks along the orbital contours (Supp. Mat. 1C) using Avizo v.2020.3. Semi-landmarks were projected onto the skull surface using a thin-plate spline deformation [8] and slid [9, 10].

### Additional descriptors of the forehead, orbits, and skull

The orbital landmarks (LM) were used to obtain linear measurements: inter-orbital distance (LM1-2, DIST), orbital height (average of LM3-7 and LM4-8), orbital width (average of LM1-5 and LM2-6), and the mean height/width ratio (RATIO). The cephalic index (CI) was computed as the ratio between the maximum width and length of the skull. The inter-frontal angle (IFA), defined by the projection of the most anterior point of the skull onto the plane parallel to the Frankfurt plane and passing through the two supra-orbital notches, was calculated [11].

### Frontal curvature

After smoothing [12], two first principal curvatures K1 and K2 were obtained as the first and second order derivatives at each vertex of the mesh in relation to its neighborhood. K1 accounted for convexities and K2 accounted for concavities (Supp. Mat. 3). The Area Of Interest (AOI) corresponding to the area between the glabella and the most anterior point of the anterior fontanelle was sectioned into 90 orthogonal slices; the mean curvature on each section was computed and then plotted for each subject and numbered from 0 (glabella) to 90 (anterior fontanelle) before being compared between groups. Difference in frontal curvature between each group pair was considered significant when the confidence interval was greater or lower than 0. Curvature values corresponding to the sections that significantly differed between groups were averaged and referred to as the mean frontal curvature (CURV).

### Statistical analyses

3D coordinates were aligned using Procrustes superimposition [9], enabling skull size and orientation standardization. Principal Component Analyses (PCA) were performed on the Procrustes coordinates (*prcomp* [10]). Differences in 3D skull morphology were screened using non-parametric multivariate analyses of variance including residual randomization (MANOVAs with permutation) with the scores of the individuals projected onto PCA axes (PC scores) cumulatively explaining 95% of total variance as the set of dependent variables and the group of patients and age as explanatory variables. Similarly, univariate testing was performed by considering axes separately. Permutation tests were performed with *procD.lm*, *geomorph* [13–15]. Pairwise permutation tests were performed [16]. When a univariate permutation test revealed a significant relationship between PC scores and age, the pairwise procedure tested whether PC scores residuals (obtained after a non-parametric regression on age) differed within each pair of groups.

The 50 orbital semi-landmarks were aligned using Procrustes superimposition. PCA was computed on the Procrustes coordinates to explore the variation in orbital shape corrected for size and age. Permutation tests were performed on the PC scores cumulatively explaining 95% of the total variance.

Finally, univariate permutation and pairwise tests were used to screen for differences in the set of linear variables (CURV, IFA, CI, ICV, DIST, RATIO) between all groups.

### Diagnostic tool design

Receiver Operating Characteristic (ROC) curves were computed to test the clinical predictive power of univariate parameters (CURV, IFA, DIST, RATIO) [17–19]. An optimized threshold value for decision was proposed for each variable [20].

For multivariate datasets, we used the K-nearest neighbors (KNN) to assess predictive powers. KNN is a machine-learning procedure used to assign a new patient to a group, depending on how similar it is from its K closest neighbours in the multivariate space. The multivariate dataset was split into a subset containing 80% of all individuals, randomly chosen and used as the training dataset and a subset containing the remaining 20%, used for testing. The number of neighbors (K) required to reach a decision was determined based on the variation of the accuracy of the algorithm with K—the accuracy being the proportion of correct classification within the testing dataset. The lowest possible K value corresponding to the best accuracy was retained [21]. KNN were used to test the predictive power of three multivariate datasets containing: (1) the PC scores of the PCA describing skull shape, (2) the PC scores of the PCA describing orbit shape, or (3) a set of linear variables (age, CURV, IFA, DIST, RATIO, CI).

### Evaluation of the expert diagnosis

The variables showing the highest discrimination power between groups (age, CURV, RATIO, DIST, IFA) were combined into PCA. Axes were extracted and used for non-supervised hierarchical classification (*HCPC* function, *FactoMineR* [22]). This allowed a classification of all observations by iteratively segregating individuals sharing more similarities until building a distance-based hierarchical dendrogram. Clusters were created without a priori on the data structure by cutting the tree starting from its deepest nodes. Cluster numbers were determined by selecting the one with the higher relative loss of inertia (i(clusters n + 1)/i(cluster n)). Disparity analyses were computed from the same PCA axes to quantify the morphological variability within each group [23]. Disparity metrics were compared between groups using Wilcoxon implemented tests with Bonferroni corrections for multiple testings.

## Results

### Description of the cohort

Ninety patients with TG (219.3 ± 81.4 days; 30% of girls), 27 patients with MR (379.25 ± 224.7 days; 40.7% of girls), and 90 controls (mean age 218.7 ± 107.8 days; 51.1% of girls) were included (Supp. Mat. 4). TG male/female ratio was comparable to literature [24–26], and was significantly higher than in MR and controls. MR had higher male/female ratio than controls.

### Skull shape

**Fig. 1:**
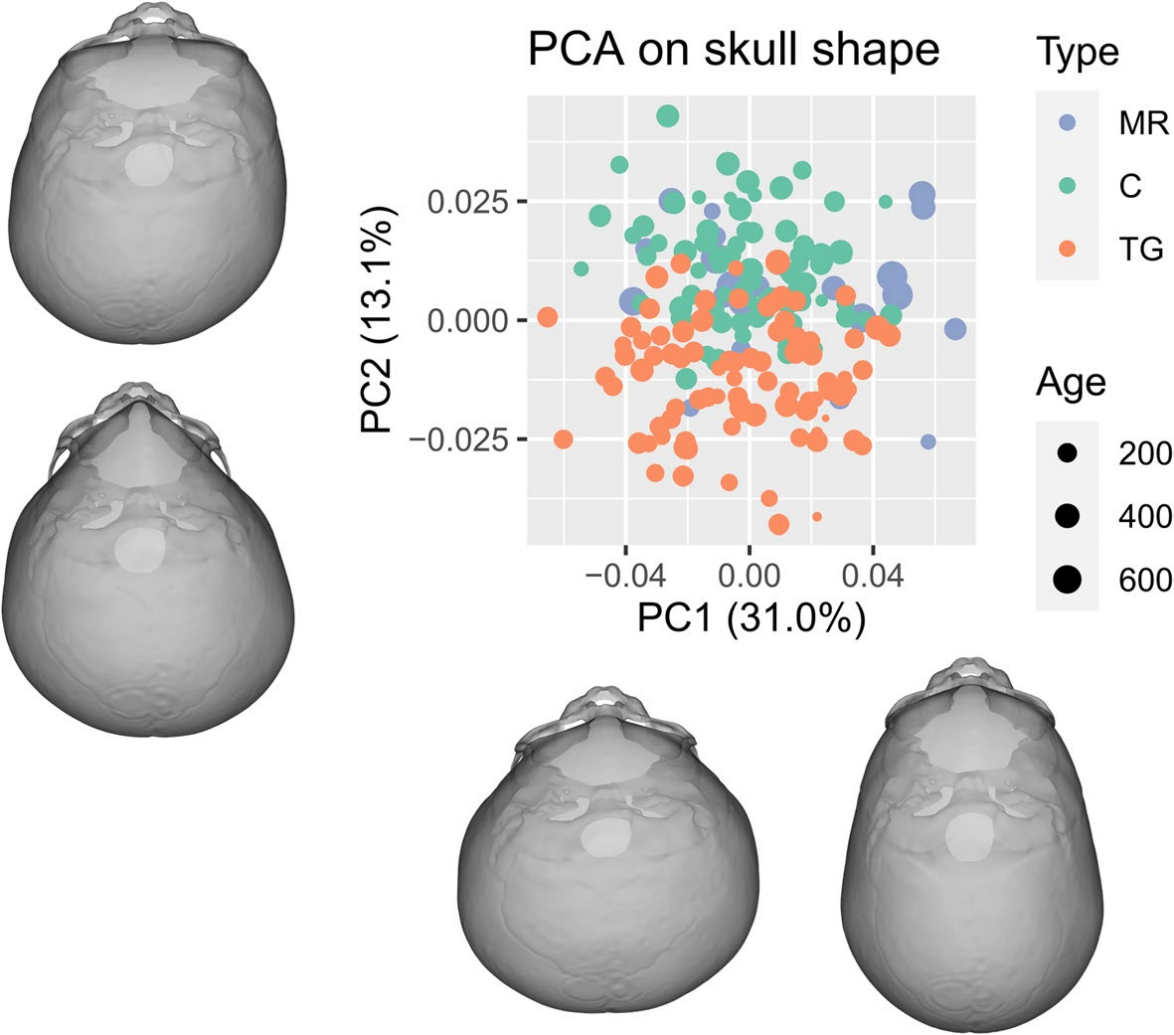
Principal Components 1 (PC1) and 2 (PC2) from the Principal Component Analysis applied on skull shapes within the cohort. 3D reconstructions represent theoretical skull shapes corresponding to positive and negative extreme values for PC1 and PC2. MR, metopic ridge; C, control; TG, trigonocephaly; PC: Principal Components. Age in days

PC1 represented 31.0% of the variance and corresponded to skull elongation and narrowing, without forehead modifications (Fig. 1). PC2 represented 13.1% of the variance and corresponded to triangular foreheads for negative values (Fig. 1). PC2, PC3 and PC4 were associated with the transversal narrowing of the anterior skull base and with an antero-posterior elongation of the foramen magnum. The multivariate permutation tests performed on the scores of the first 40 PCs (95% of the variance) showed that TG, MR and controls all differed in their 3D skull shape (Table 1). Permutation tests indicated that skull shape varied with age and that shape variation occurring during growth differed in all groups. Univariate and pairwise permutation tests showed that although PC1 only discriminated MR from TG, it discriminated all pairs of groups when accounting for age; PC2 and PC3 discriminated MR from TG and controls from TG (Table 1), while PC4 discriminated MR from controls and controls from TG.

**Table 1:** Multivariate, univariate and pairwise permutation tests screening for differences in skull shape between groups and according to age. PC: Principal Component; Df: degrees of freedom; F: F-statistic; R2: coefficient of correlation; P: *P*-value; MR: metopic ridge; C: normal; TG: trigonocephaly. Bold: *P*-value < 0.05

		All PCs	PC1	PC2	PC3	PC4	
Permutation tests	Group	2	2	2	2	2	Df
		15.72	3.95	69.88	23.26	27.88	F
		0.126	0.038	0.395	0.178	0.143	R^2^
		** < 0,001**	**0.020**	**0.001**	**0.001**	**0.001**	*P*
	Age	1	1	1	1	1	Df
		7.94	0.59	3.2	9.03	72.32	F
		0.032	0.003	0.009	0.034	0.185	R^2^
		** < 0,001**	0.473	0.087	**0.002**	**0.001**	*P*
	Group: Age	2	2	2	2	2	Df
		3.24	0.06	3.81	0.01	15.31	F
		0.026	0.001	0.022	0.013	0.153	R^2^
		**0.003**	0.946	**0.030**	0.148	**0.001**	*P*
Pairwise tests	MR vs C		0.696	0.192	1	**0.002**	*P*
	MR vs TG		0.056	** < 0,001**	** < 0,001**	1	
	C vs TG		0.119	** < 0,001**	** < 0,001**	** < 0,001**	
	MR		-67.9	100	-140	94.3	Means
	C		19.7	192	-96.3	-109	
	TG		-81.3	-220	155	116	

### Orbital shape

**Fig. 2:**
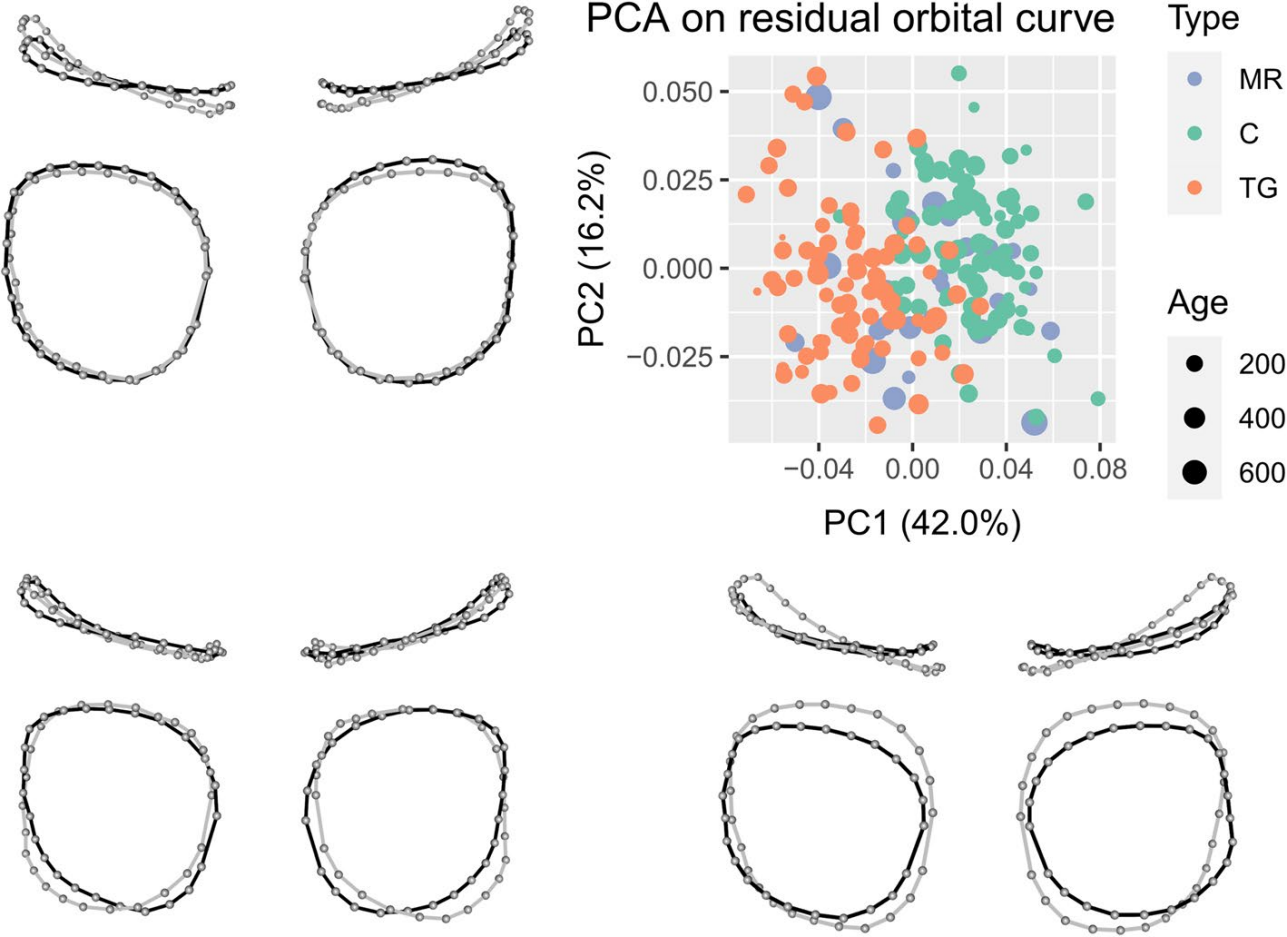
Principal Components 1 (PC1) and 2 (PC2) from the Principal Component Analysis applied on orbital shape within the cohort, representing theoretical orbital curves corresponding to each extreme PC values with minimal values in grey and maximal values in black; superior and frontal views. MR, metopic ridge; C, normal; TG, trigonocephaly. Age in days

**Fig. 3:**
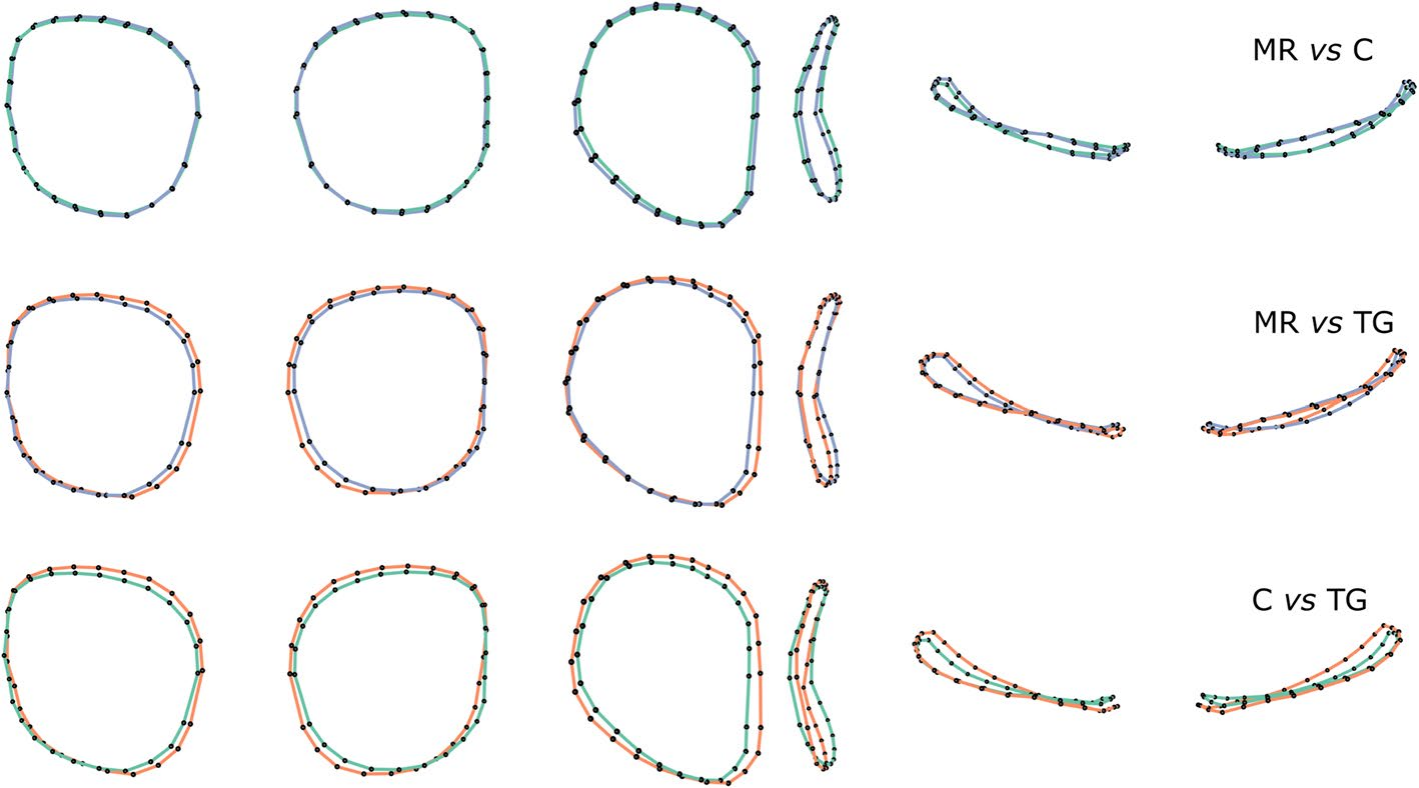
Mean orbital shapes within each group generated from the first 4 Principal Components. From left to right: frontal, right lateral, and superior views. MR: metopic ridge; C: control; TG: trigonocephaly

Negative PC1 values (42.0% of variance) corresponded to an increase in medial orbital height, and a to greater superior orbital width (Fig. 2). The medial aspect of the orbits was translated forwards, associated with lateral retrusion, inducing a more triangular outline. Negative PC2 values (16.2% of the variance) corresponded to a decrease in orbit height and a similar orientation change as previously described for PC1 (Fig. 2). The permutation tests performed on the scores of the 17 first PCs (95% of the variance) indicated that orbital shape statistically differed between all groups (Table 2). PC1 and PC4 discriminated each pair of groups. PC2 discriminated controls from TG. PC3 discriminated MR from TG and controls from TG (Table 2). Relative to controls, orbits in TG were higher and narrower, their medial aspect was moved forward, and their lateral aspect was tilted backwards. The angle formed by the two orbits thus seemed more acute in TG (Fig. 3). The orbits in MR presented a distinctive intermediate shape between controls and TG.

**Table 2:** Multivariate, univariate and pairwise permutation tests screening for differences in orbital curves between groups. PC: Principal Component; Df: degrees of freedom; F: F-statistic; R2: coefficient of correlation; P: *P*-value; MR: metopic ridge; C: normal; TG: trigonocephaly. Bold: *P*-value < 0.05

		All PCs	PC1	PC2	PC3	PC4	
Permutation tests	Group	2	2	2	2	2	Df
		34.73	111.24	5.053	10.348	14.593	F
		0.268	0.539	0.051	0.098	0.133	R^2^
		0.001	**0.001**	**0.011**	**0.001**	**0.001**	*P*
Pairwise tests	MR vs C		**0.001**	0.101	0.051	**0.014**	*P*
	MR vs TG		** < 0,001**	1	**0.001**	** < 0,001**	
	C vs TG		** < 0,001**	**0.009**	**0.024**	**0.005**

### Forehead curvature

**Fig. 4:**
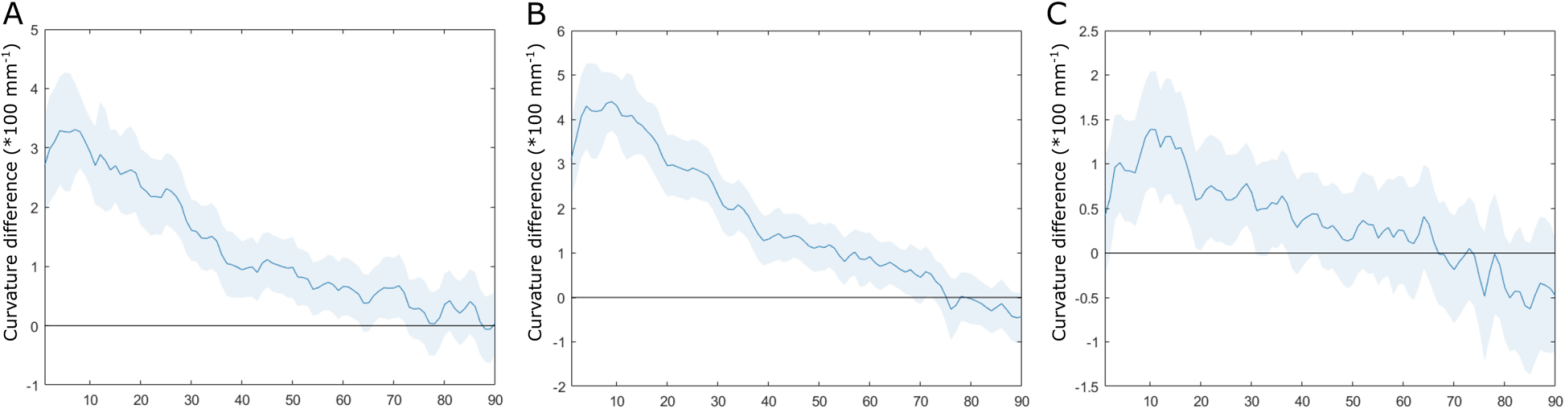
Mean curvature difference and confidence interval of the differences compared to 0 A: between trigonocephaly and metopic ridges; B: between trigonocephaly and controls; C: between metopic ridges and controls

Frontal curvature in MR and controls was nearly constant from the glabella to the anterior fontanelle (Supp. Mat. 5). Curvature was greater near the glabella in TG than in MR or controls. Frontal curvature differed in the interval including sections 1 to 68 for TG and MR, in the interval including sections 1 to 63 for TG and controls, and in the interval between sections 2 and 31 for MR and controls (Fig. 4). CURV was thus calculated as the average curvature from sections 2 to 31 in all groups.

### Univariate morphometric parameters

The univariate permutation tests performed on CI, CURV, DIST, ICV, IFA and RATIO showed that CURV, DIST and IFA significantly differed between all groups even when accounting for age (Table 3). ICV differed between MR and controls, and between controls and TG when accounting for age. RATIO differed between MR and TG and between controls and TG. When accounting for age, CURV was the greatest in TG and the lowest in controls; DIST was the greatest in controls and the lowest in TG; the most obtuse IFA was found in controls, and the most acute in TG; TG and MR patients had in average greater ICV than controls; and MR had greater RATIO than controls (Table 3).

**Table 3:** Univariate and pairwise permutation tests screening for differences in single variables (CI, CURV, DIST, ICV, IFA, RATIO) between groups, according to age. Df: degrees of freedom; F: F-statistic; R2: coefficient of correlation; P: *P*-value; MR: metopic ridge; C: normal; TG: trigonocephaly patients. Bold: *P*-value < 0.05. CI: Cephalic Index; CURV: mean frontal curvature, DIST: inter-orbital distance; ICV: Intra-Cranial Volume; IFA: Interfrontal Angle; RATIO: orbital height/width ratio)

		CI	CURV	DIST	ICV	IFA	RATIO	
Permutation tests	Group	2	2	2	2	2	2	Df
		2.406	236.72	112.46	11.706	98.88	11.63	F
		0.025	0.703	0.526	0.062	0.485	0.107	R^2^
		0.101	**0.001**	**0.001**	**0.001**	**0.001**	**0.001**	*P*
	Age	1	1	1	1	1	1	Df
		0.15	0.18	6.71	144.99	5.08	5.21	F
		0.001	0.001	0.016	0.384	0.012	0.024	R^2^
		0.715	0.650	**0.019**	**0.001**	**0.028**	**0.019**	*P*
	Group: Age	2	2	2	2	2	2	Df
		0.037	0.474	4.45	6.769	3.06	0.04	F
		0.001	0.001	0.021	0.036	0.015	0.001	R^2^
		0.963	0.613	**0.014**	**0.002**	0.050	0.960	*P*
Pairwise tests	MR vs C	0.473	** < 0,001**	** < 0,001**	**0.005**	**0.005**	**0.132**	*P*
	MR vs TG	0.599	** < 0,001**	** < 0,001**	0.264	** < 0,001**	** < 0,001**	
	C vs TG	0.128	** < 0,001**	** < 0,001**	**0.022**	** < 0,001**	**0.002**	
	MR	-68.3	5.66	15.4	946	118	0.920	Means
	C	-26.7	4.55	16.9	863	130	0.886	
	TG	26.8	8.06	13.8	922	109	0.866	

Spearman’s rank correlation tests performed against age showed that CI, CURV, and RATIO did not correlate with age in any group; DIST increased with age in TG only; ICV increased with age in all three groups, but the correlation slope was the greatest in controls and the lowest in TG (Table 4), suggesting that ICV increased more slowly in MR and TG; IFA increased with age only in controls.

**Table 4:** Spearman’s rank correlation tests screening for a relationship between each single variable (CI, CURV, DIST, ICV, IFA, RATIO) and age. S: test statistics; Rho: estimate of association; P: *P*-value. Bold: *P*-value < 0.05. CI: Cephalic Index; CURV: mean frontal curvature, DIST: inter-orbital distance; ICV: Intra-Cranial Volume; IFA: Interfrontal Angle; RATIO: orbital height/width ratio)

	CI	CURV	DIST	ICV	IFA	RATIO	
Controls	94,177	125,343	75,979	19,681	163,917	101,122	S
	0.012	-0.038	0.203	0.832	-0.349	-0.061	Rho
	0.917	0.766	0.066	** < 0,001**	** < 0,001**	0.582	*P*
Metopic ridge	3244.9	2497.5	3127.2	449.76	3350.5	2497	S
	-0.109	0.146	-0.069	0.783	-0.145	0.146	Rho
	0.595	0.476	0.737	** < 0,001**	0.478	0.476	*P*
Trigonocephaly	102,621	117,992	63,921	39,254	112,944	94,489	S
	-0.039	-0.004	0.353	0.666	0.039	0.043	Rho
	0.725	0.968	**0.001**	** < 0,001**	0.719	0.695	*P*

A two-way analysis of variance with the residual ICV as explained variable and sex and group as dependent variables revealed that residual the ICV depended on sex (*R*^2^ = 0.11; *F* = 13.28; *p* = 0.001) and group (*R*^2^ = 0.08; *F* = 18.20; *p* = 0.001). The interaction between sex and group was not significant (*R*^2^ < 0.01; *F* = 0.13; *p* = 0.894), suggesting that the differences in ICV between groups were not driven by age differences.

## Screening for diagnostic features

### Univariate predictors / ROC Curves

**Fig. 5:**
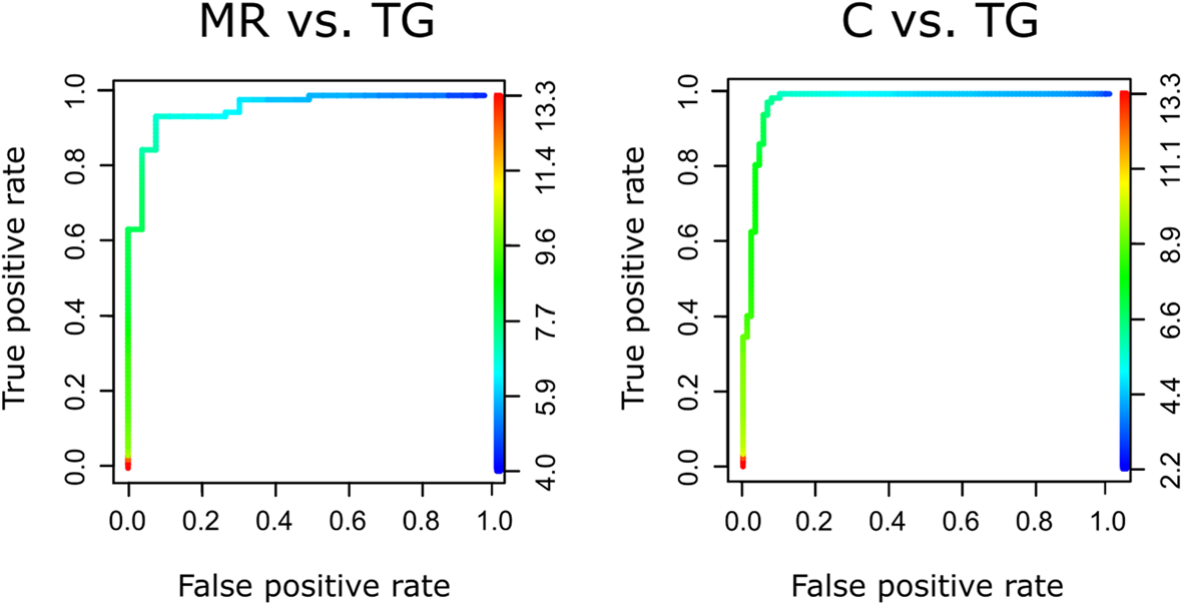
Receiver Operating Characteristic (ROC) curves testing the variable CURV (mean frontal curvature) to predict a binary outcome. Color gradient: range of predictor values. CURV in mm^−1^. MR: metopic ridge; C: normal; TG: trigonocephaly

ROC analyses indicated that CURV was a quantitative predictor of groups (Table 5, Fig. 5). CURV enabled the recognition of controls from TG (sensitivity and specificity > 93%) and MR from TG (sensitivity and specificity > 92%), but not MR from controls (sensitivity 6%). To differentiate controls from TG and MR from TG, the results suggested threshold CURV values of 6.179 and 6.721, respectively. On the contrary, DIST, IFA and RATIO were poor predictors (Table 5, Supp. Mat. 6).

**Table 5:** Receiver Operating Characteristics (ROC) estimating the ability of a quantitative variable (CURV, DIST, IFA, RATIO) to predict a binary outcome. MR: metopic ridge; C: normal; TG: trigonocephaly. Bold: sensitivity and specificity values > 0.90. CURV: mean frontal curvature, DIST: inter-orbital distance; IFA: Interfrontal Angle; RATIO: orbital height/width ratio)

	CURV	DIST	IFA	RATIO	
MR vs TG	**0.944**	0.167	0.337	0.667	Sensitivity
	**0.926**	0.5	0.423	0.231	Specificity
	6.721	15.27	112.69	0.846	Cutoff
MR vs C	0.056	0.699	0.689	0.675	Sensitivity
	**0.926**	0.731	0.654	0.308	Specificity
	7.009	16.19	123.54	0.873	Cutoff
C vs TG	**0.978**	0.167	0.191	0.369	Sensitivity
	**0.933**	0.5	0.176	0.542	Specificity
	6.179	17.51	109.27	0.888	Cutoff

### Multivariate predictors / KNN analyses

KNN provided satisfying classification results when considering the PC scores of the PCA describing skull shape (accuracy = 90%), with 100% of controls and TG correctly assigned, but with a poor recognition of MR (50%). When considering the PC scores associated with orbital shape, classification results were less satisfying (accuracy = 82%). When considering a set of 6 univariate measures (AGE, CURV, IFA, DIST, RATIO, CI), classification results reached 100% accuracy.

### Non-supervised hierarchical classification

**Fig. 6:**
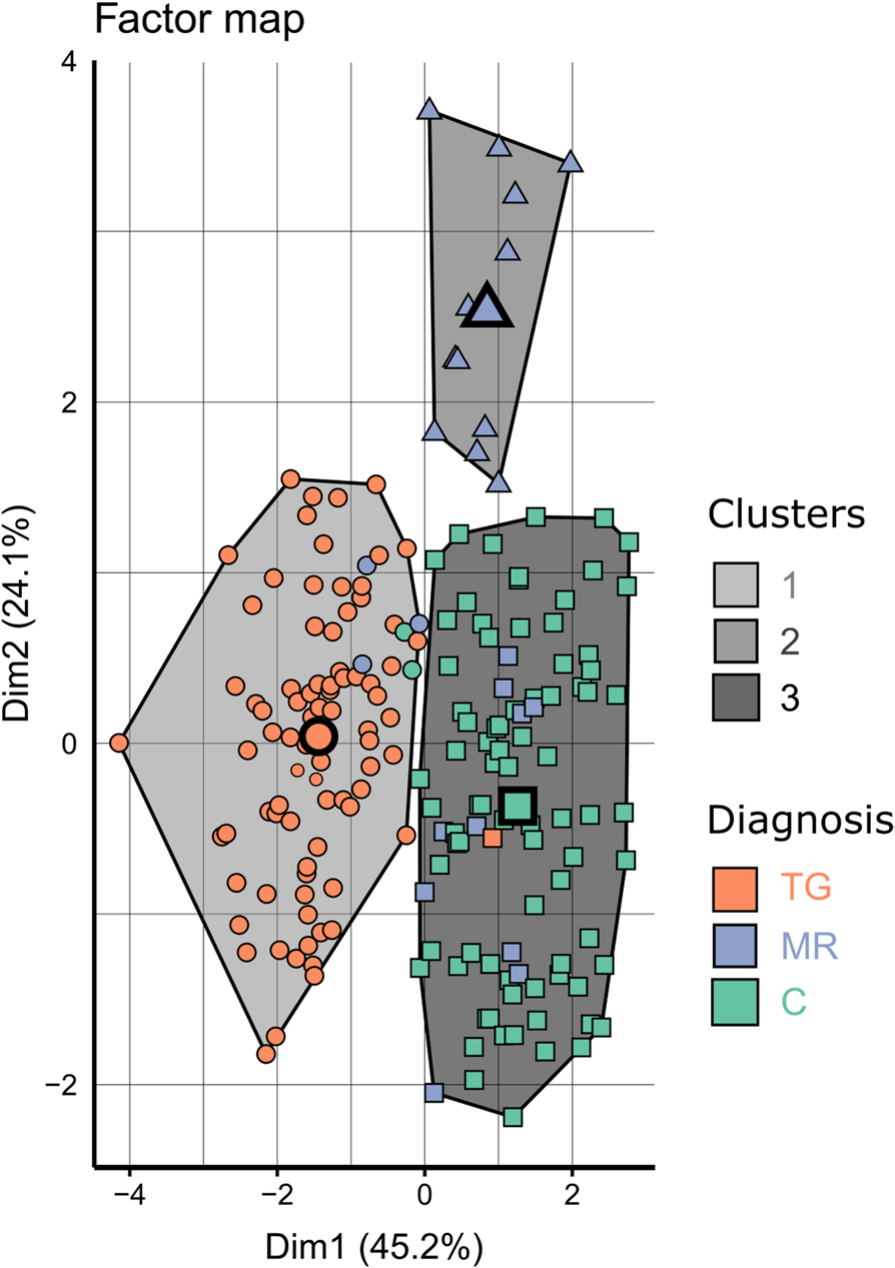
The two first axes (Dim1 and Dim2) of the Principal Component Analysis used to perform the Hierarchical Classification on Principal Components. These two axes created a two dimensional morphological space where each point represented a patient, allowing to appreciate the relative consistency between non a priori clustering and diagnosis

**Fig. 7:**
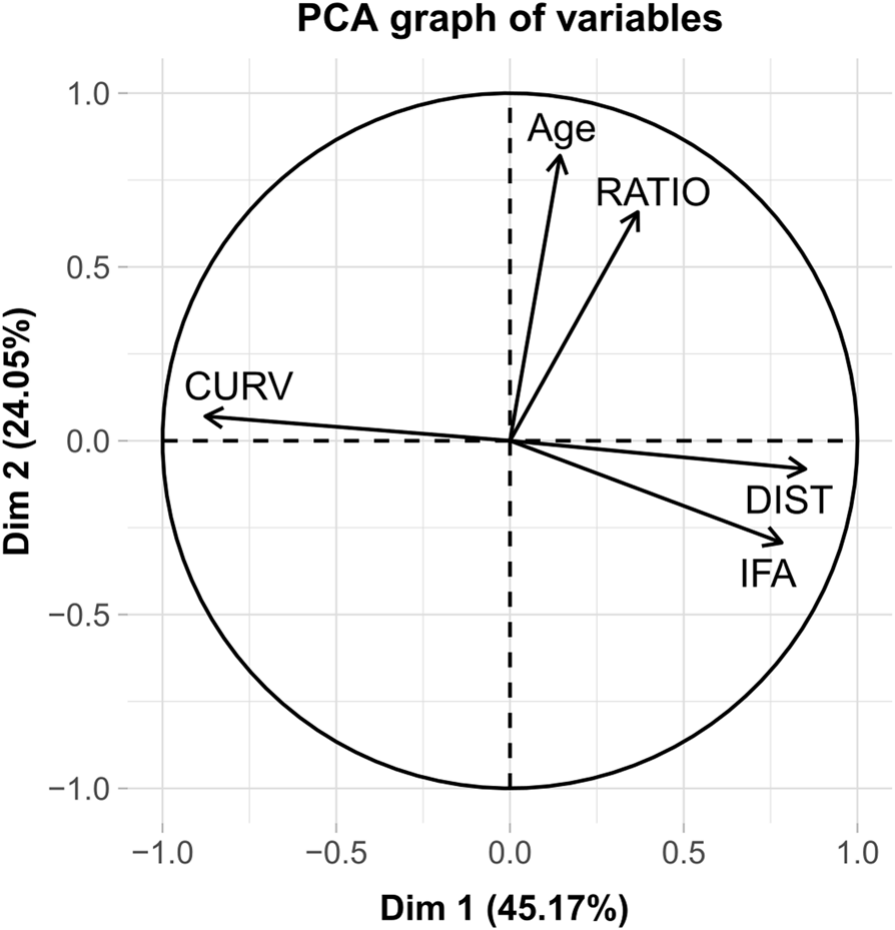
Correlation circle representing the morphometric variables underlying the distribution of the subjects in the morphological space created by the first two Principal Components. The circle suggests that patients included in Cluster 1 (see Fig. 6) showed a greater frontal curvature (CURV); patients in Cluster 2 were older and presented a greater orbital ratio (RATIO); patients in Cluster 3 had a more obtuse interfrontal angle (IFA) and a greater interorbital distance (DIST)

Clusters 1, 2 and 3 were highlighted by the classification process, and corresponded to the number of clusters before the gain of inertia started to decline (Supp. Mat. 7a-b). These three clusters were distinct in the morphospace (Fig. 6). The circle of correlations (Fig. 7) indicated that cluster 1 mostly differed by showing greater CURV, cluster 2 by containing older patients with a greater RATIO, and cluster 3 by showing greater DIST and IFA. A confusion matrix was produced to confront the initial diagnosis (C vs MR vs TG) with HCPC clusters. Clusters 1 and 3 almost perfectly matched TG and controls, respectively. Cluster 2 also matched MR, to a lesser extent. Interestingly, patients initially diagnosed with MR that were not grouped into cluster 2 were nearly all grouped in cluster 3, corresponding to controls (Table 6). It appeared that discrepancy between diagnosis and clustering might be influenced by age (Supp. Mat. 7a-b, 8). More specifically, disparity, meaning the overall morphological variability or spreading, was significantly the highest in MR, and the lowest in TG (Supp. Mat. 8). When accounting for age, disparity was higher at younger ages (below 10 months) in controls and TG, but not in MR (Supp. Mat. 9).

**Table 6:** Confusion matrix confronting the original clinical diagnosis (metopic ridge: MR; controls: C; trigonocephaly: TG) with the clustering proposed by the Hierarchical Classification on Principal Components (HCPC). The matrix lists the correspondences between the diagnosis by an expert and the prediction without a priori based on quantitative morphological traits

	Original diagnosis			
		MR	C	TG
Predicted group	1	3	2	83
	2	12	0	0
	3	11	81	1

## Discussion

### Towards a diagnostic tool for ‘triangular foreheads’

‘Triangular foreheads’ represent a significant number of patients managed by craniofacial teams. Uncertain early differential diagnosis between TG and MR leads to stress for parents before their referral to specialized departments. Several studies have proposed objective criteria for distinguishing TG from MR [2, 3, 27]. By combining morphometric parameters (age, IFA, DIST, RATIO, CI) and innovative approaches (CURV), we confirmed that these two conditions can be distinguished reliably. Furthermore, without assumptions, our criteria overlap the initial differential diagnosis from experts. Our approach is based on CT-scans as we assessed a historical cohort from a national French reference center. Patients with single suture craniosynostoses now benefit from MRI and/or 3D photography. Patients with metopic ridges do not require radiological assessment: the CT-scans included in this study were performed before referral, because of differential diagnosis doubts with TG. Our MR sample may thus represent a sub-group raising sufficient diagnostic issues to justify radiology and referral. This sub-group would then be particularly relevant for assessing the potentiality of a differential diagnosis algorithm. The initial clinical diagnosis in our center was supported by quantification: our results suggest that the tool we developed could be interesting to avoid CT-scans in difficult MR cases. More generally, we suggest that referral to expert centers should be the first option before performing a CT-scan in MR patients with suspected TG.

The tool we designed requires clinical validation. While CT-scans were previously used routinely to assess forehead shape in TG, we now rely on MRI for initial assessment. Automatic segmentation of the bone on MRI is not straightforward and applying our method to MRI data requires further development [28, 29], while 3D photogrammetry provides data that could be directly processed for surface quantification [30].

### Expert opinion vs quantification

Our quantitative approach generated 3 clusters without a priori, that almost perfectly corresponded to the three groups defined by experts. The only misattribution observed was 11/27 MR grouped with controls. Almost no (3/27) MR was grouped with TG, suggesting that the main unsolved diagnostic question was controls vs MR, which does not represent a major clinical issue. Moreover, the patients that were misattributed were usually the youngest (< 10 months). After 11 months, the correspondence between the MR cluster and MR diagnosed by experts improved, suggesting that early diagnosis of MR was specifically difficult. Interestingly, the performance of the KNN algorithm that earlier provided the best accuracy (100%) decreased to 93% when considering only subjects under 10 months, due to a less accurate prediction of the MR patients who were grouped with controls in most misattribution cases (Supp. Mat. 9).

The influence of age on diagnostic accuracy was supported by the analyses on morphological disparity: craniofacial morphology was less stereotyped at younger ages in all groups except in MR; and morphological stereotypy was maximal in TG. This suggests that the morphological traits leading to MR expert diagnosis were less constant than for TG, especially before 10 months of age. We also report that disparity tended to decrease with age, which most probably reflected the influence of homogenous external factors (brain growth, orbital growth) on skull growth, that took over initial congenital shape specificities.

More generally, the fact that a classification without a priori produced three distinct clusters matching the expert opinions suggests that clinical impression is at least partly driven by the visual assessment of orbital proportions, hypotelorism and frontal curvature. This fact supports the value of our quantitative approach, based on a preliminary clinical assessment, considered here as a gold standard.

### Increased intracranial volume in trigonocephaly

Decreased ICV in TG has been reported [31–33], as well as conserved volumes in moderate cases (41/58 cases) and decreased volumes in 18/58 severe cases defined by IFA < 123° [34]. Surprisingly, we found increased ICV in TG and MR relative to age, independently of sex, compared to the normal cohort, with reasonable reliability as we report the largest series in the literature (10 to 74 patients to date in previous series [31–39]). In MR, a single evaluation reported larger ICV values than in TG [39], while we report no difference of ICV relative to age between TG and MR. This finding raises the issue of the origins of potential increased ICP in TG. Recent studies focusing on brain perfusion using Arterial Spin Labeling (ASL) have reported specific frontal decreases in blood flow, suggesting that brain compression could be regional and functionally significant despite global ICV increase [37, 40, 41].

### New morphometric parameters in trigonocephaly

Several methods for the estimation of the interfrontal angle have been proposed: metopic severity index [42], interfrontal divergence angle [43], and IFA [11, 44]. IFA was proven to distinguish TG from controls with sensitivity and specificity greater than 94% [11]. Nevertheless, IFA accuracy in MR diagnosis and its evolution with age were unknown. We report that IFA is stable in time in TG and MR and decreases with age in controls. This result suggests that the midline forehead shape is most probably determined by prenatal metopic fusion both in TG and MR. Severe forms in early childhood are not likely to normalize spontaneously. On the contrary, moderate forms will not evolve into more severe phenotypes. In this context, the rare forms with intermediate phenotypes raise tricky issues and should ideally benefit from functional brain imaging that could potentially indicate surgery in case of lowered blood-flow. Based on our results, functional approaches seem to be the main solution for future rational indications in trigonocephaly surgery, and most probably in the surgical management of other single-suture craniosynostoses.

Hypotelorism is part of the diagnostic triad of TG [45], while there is an intermediate inter-orbital dysmorphology in MR [3]. We confirm that the inter-orbital distance is lowest in TG and intermediate in MR. Orbital shape in TG is modified: medial orbits are moved forward, and lateral orbits tilted backwards.

## Conclusion

TG and MR can be distinguished based on quantitative criteria that match expert opinions from a large reference center. We suggest that these conditions are distinct clinical entities, with specific characteristics such as interfrontal curvature and orbital shape anomalies. We provide bases for a diagnostic tool for ‘triangular foreheads’ intended for local centers, in order to discuss referral to reference craniofacial centers.

### Supplementary Information

Supplementary Material 1. A and B. Craniofacial landmarks used for morphological assessment: left and right supra-orbital notch, left and right infra-orbital foramen, left and right porion, opisthion and lambda. C. Orbital landmarks used for morphometric assessment: 8 anatomical landmarks (red dots) and 50 semi-landmarks along curves (green dots) were placed on each subject – (1, 2) fronto-maxillary junction; (3, 4) middle of the supraorbital bar; (5, 6) ectoconchion; (7, 8) zygo-orbitale.

Supplementary Material 2. From left to right: wrap template mesh, initial geometry for a patient, and wrapped skull for the same patient.

Supplementary Material 3. Normal skull with a colormap associated to the curvature K1 and K2 expressed in mm-^1^. Positive curvature values accounted for concavities and negative curvature values corresponded to convex areas.

Supplementary Material 4. Mean age, cephalic index, intracranial volume (ICV) and interfrontal angle (IFA) for each group. MR: metopic ridge; C: control; TG: trigonocephaly; f: female; m: male; Sd: standard deviation.

Supplementary Material 5. Curvature analysis from the section near glabella (section 0) to the section near the anterior fontanelle (90). A: trigonocephaly, B: metopic ridge, C: controls. Solid color line: mean value with standard error.

Supplementary Material 6. Receiver Operating Characteristic (ROC) curves testing the ability of univariate variables (IFA, CURV) to predict a binary outcome. Color gradient: range of predictor values. IFA (interfrontal angle) in degrees, CURV (mean frontal curvature) in mm-^1^. MR: metopic ridge; C: control; TG: trigonocephaly.

Supplementary Material 7. a and b. Hierarchical dendrogram showing the similarity-based relationships between all included subjects. Three clusters were automatically created without a priori by separating the deepest branches in the tree to optimize the relative loss of inertia. Colors display the original diagnosis proposed by the experts (green: controls, blue: metopic ridge, orange: trigonocephaly). When diagnosis did not match the cluster prediction, the patient was marked with a triangle, and age in days was indicated.

Supplementary Material 8. Comparison of morphological disparity metrics between diagnosis groups (MR, metopic ridge; C, control; TG, trigonocephaly) (on the left), and depending on age (+: after 10 months of age; -: before 10 months of age) (on the right). All subsets were significantly different (all p<0.05) from each other, except between MR+ and MR- (red cross).

Supplementary Material 9. Dependency of prediction accuracy to age (<10 months vs > 10 months). Younger patients were most often misattributed than patients >10 months of age, even though the overall classification skills of the algorithms were satisfactory (MR, metopic ridge; C, control; TG, trigonocephaly).

## Data Availability

The datasets generated and/or analysed during the current study are not publicly available but are available from the corresponding author on reasonable request.
