# Supplementary figures and images for: New diagnostic criteria for metopic ridges and trigonocephaly: a 3D geometric approach
Source: Orphanet J Rare Dis. 2024 May 18;19:204. doi: 10.1186/s13023-024-03197-8 (PMC11102612; doi:10.1186/s13023-024-03197-8)

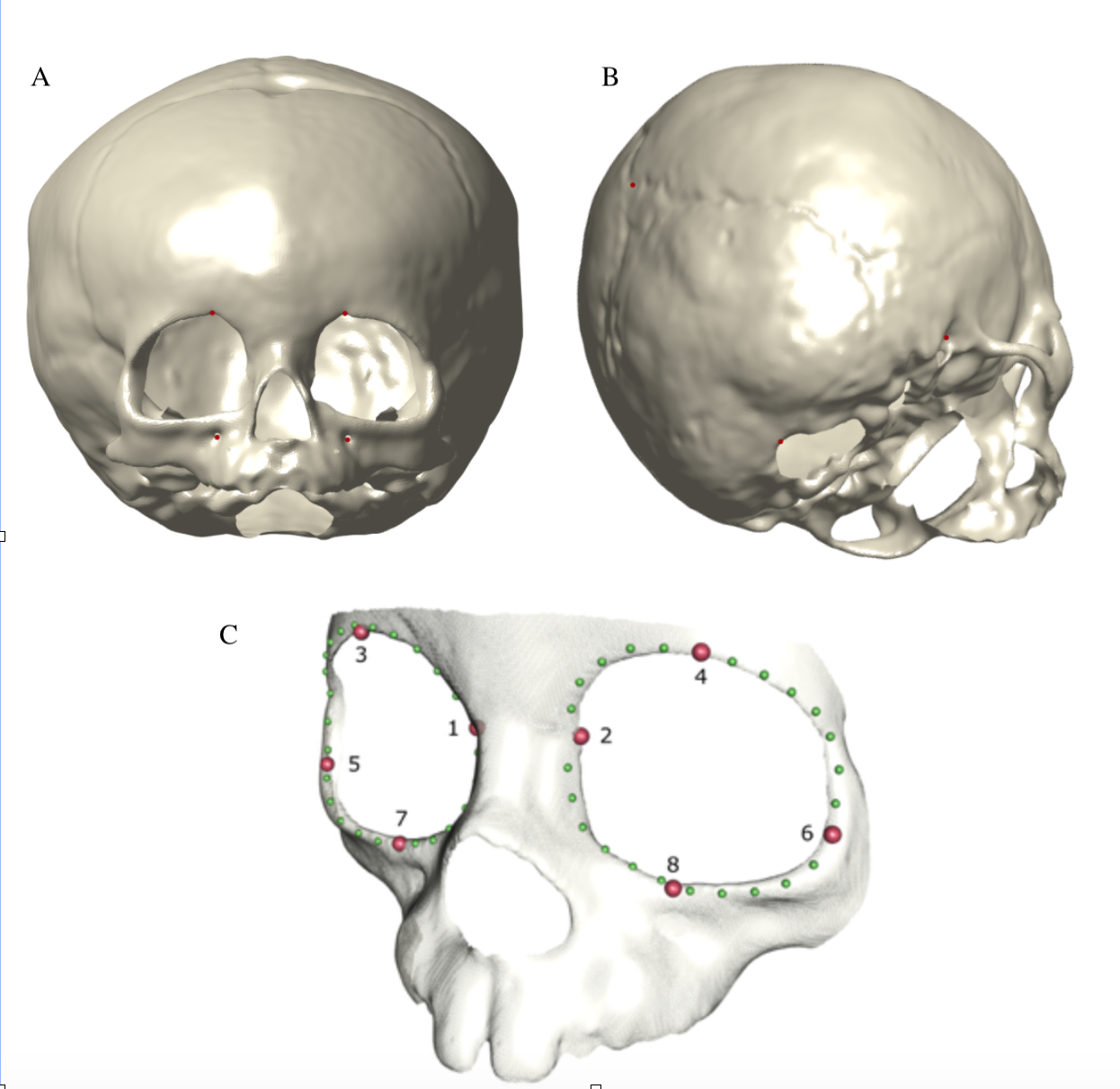

Supplement: Supplementary file 1 — Supplementary Material 1. A and B. Craniofacial landmarks used for morphological assessment: left and right supra-orbital notch, left and right infra-orbital foramen, left and right porion, opisthion and lambda. C. Orbital landmarks used for morphometric assessment: 8 anatomical landmarks (red dots) and 50 semi-landmarks along curves (green dots) were placed on each subject – (1, 2) fronto-maxillary junction; (3, 4) middle of the supraorbital bar; (5, 6) ectoconchion; (7, 8) zygo-orbitale. [file 13023_2024_3197_MOESM1_ESM.png]

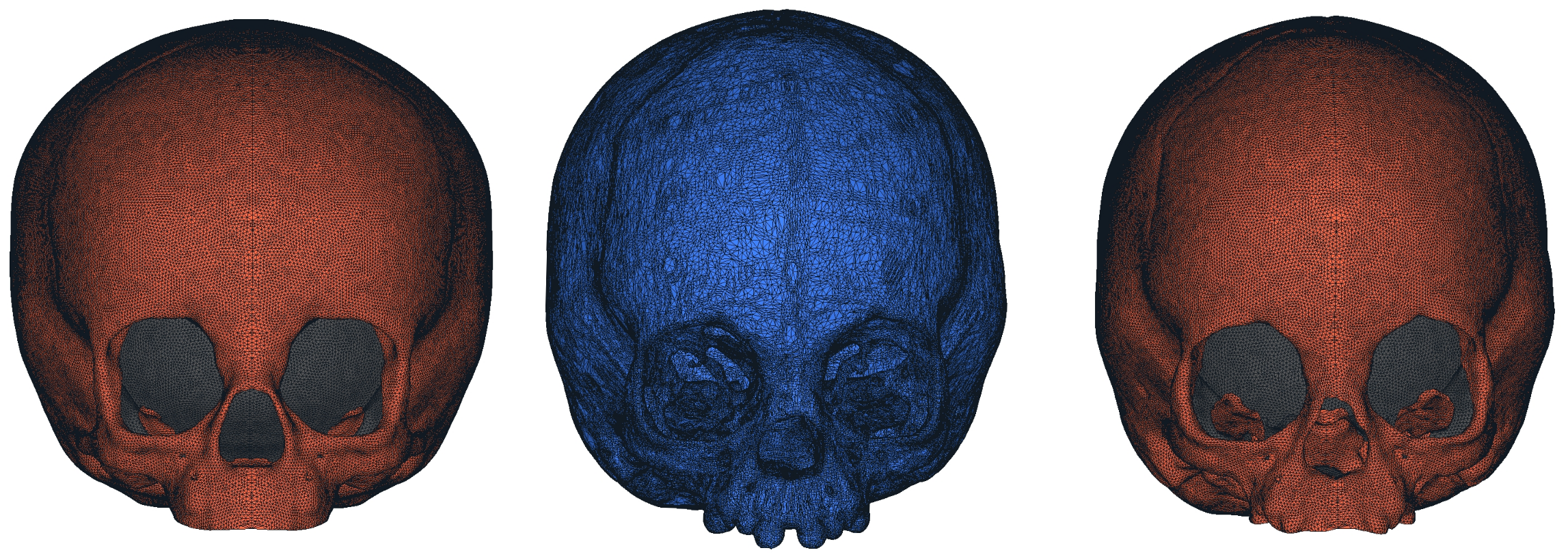

Supplement: Supplementary file 2 — Supplementary Material 2. From left to right: wrap template mesh, initial geometry for a patient, and wrapped skull for the same patient. [file 13023_2024_3197_MOESM2_ESM.png]

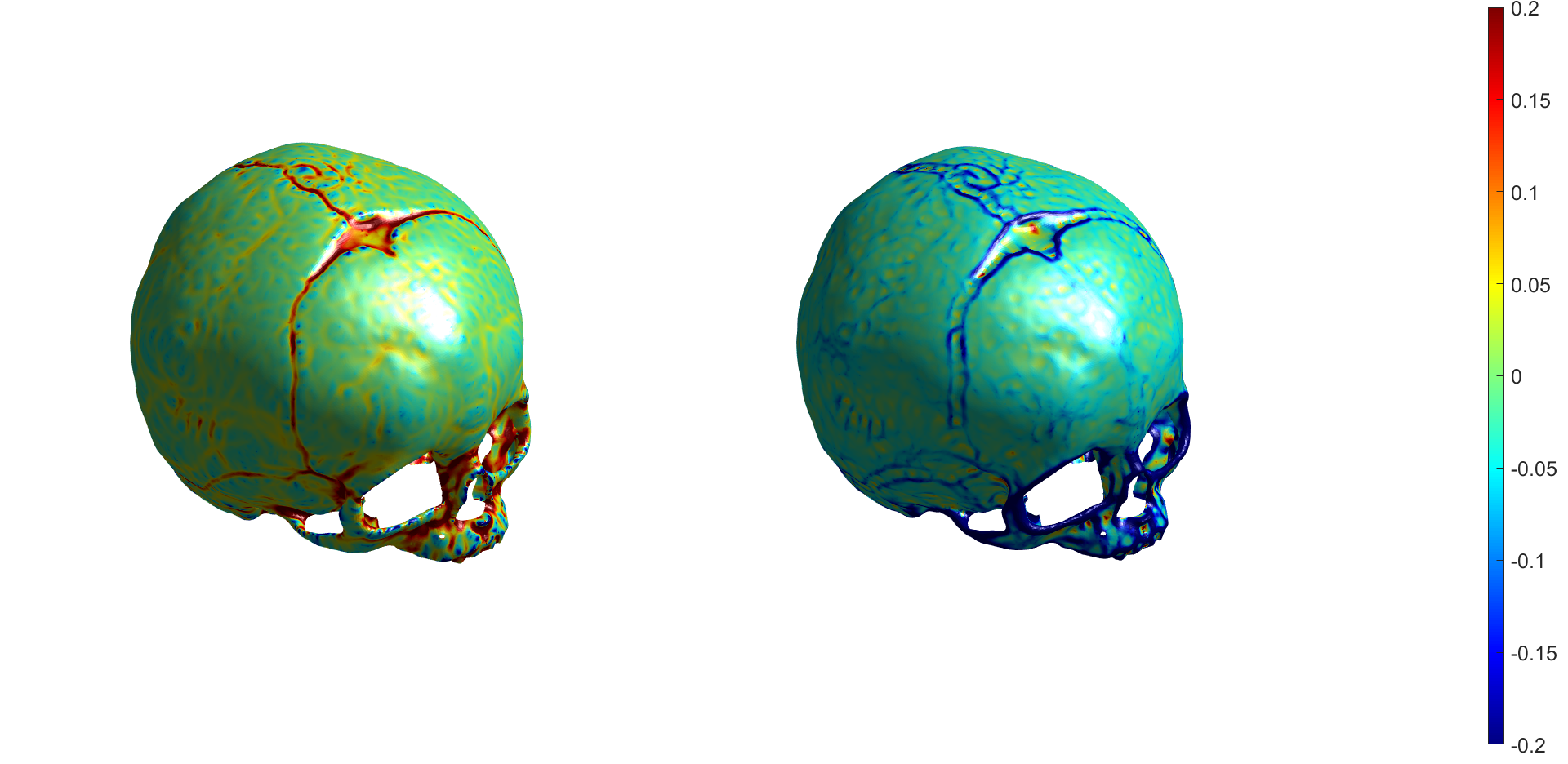

Supplement: Supplementary file 3 — Supplementary Material 3. Normal skull with a colormap associated to the curvature K1 and K2 expressed in mm-1. Positive curvature values accounted for concavities and negative curvature values corresponded to convex areas. [file 13023_2024_3197_MOESM3_ESM.png]

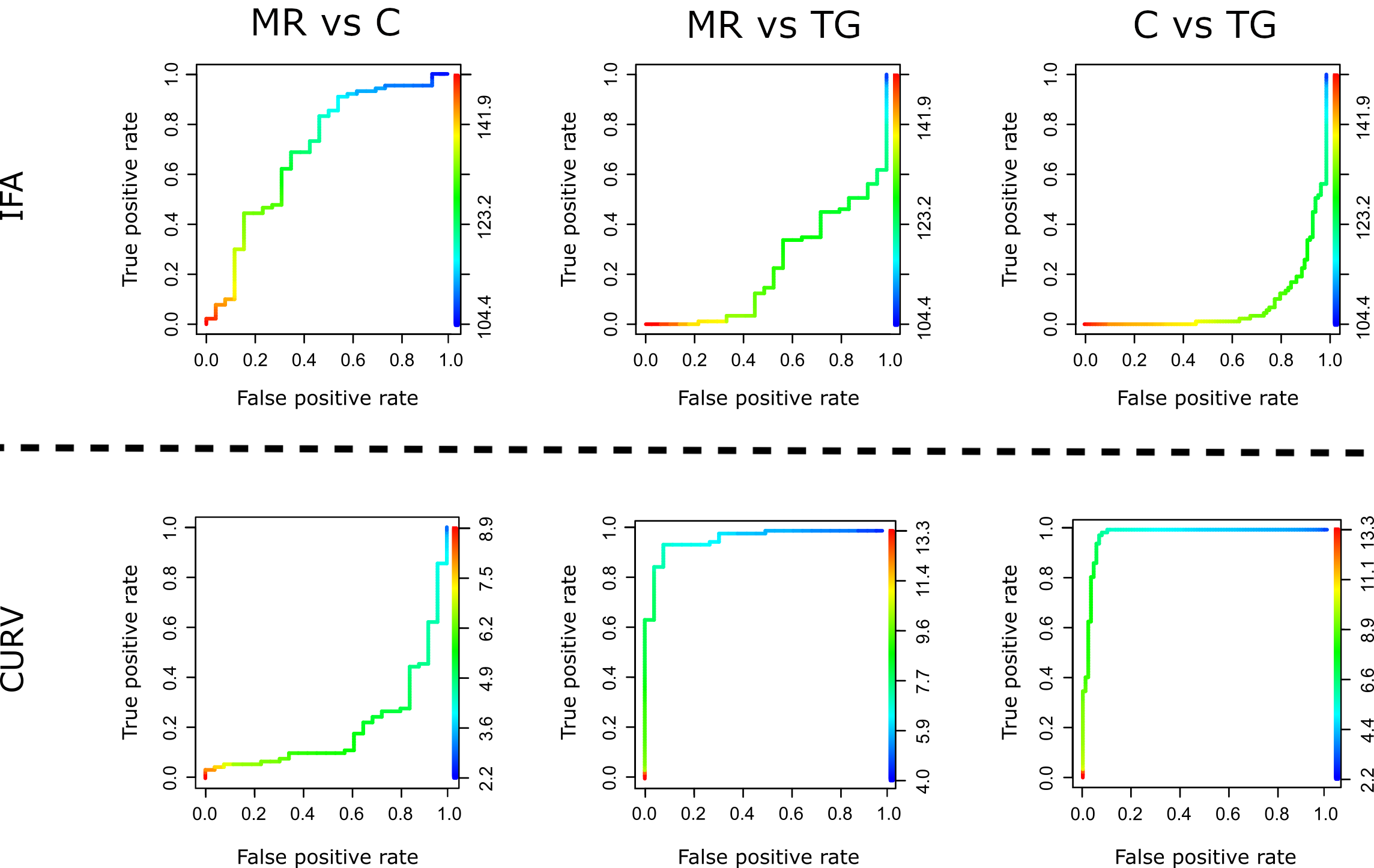

Supplement: Supplementary file 6 — Supplementary Material 6. Receiver Operating Characteristic (ROC) curves testing the ability of univariate variables (IFA, CURV) to predict a binary outcome. Color gradient: range of predictor values. IFA (interfrontal angle) in degrees, CURV (mean frontal curvature) in mm-1. MR: metopic ridge; C: control; TG: trigonocephaly. [file 13023_2024_3197_MOESM6_ESM.png]

Figure 7a

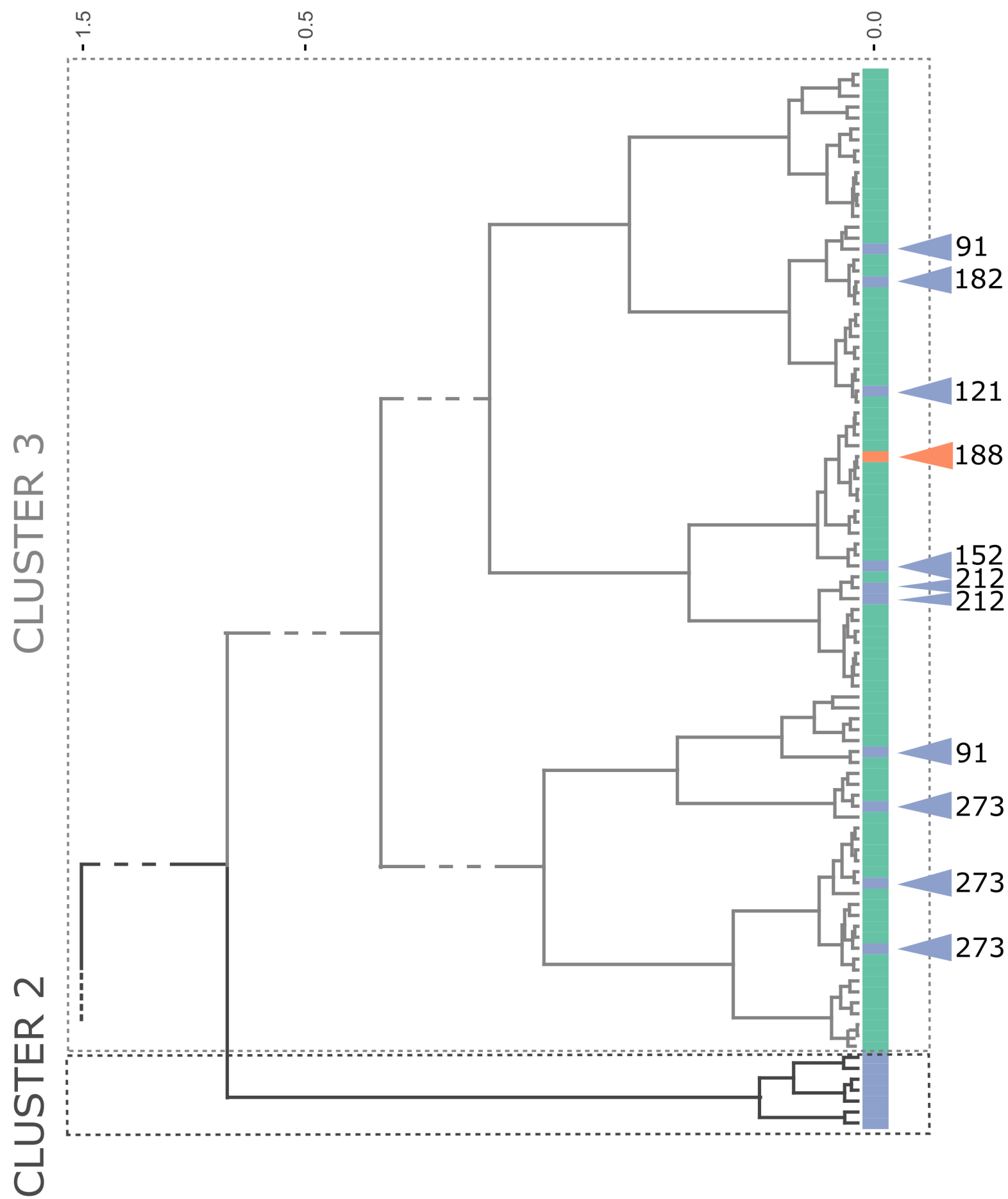

Figure 7b

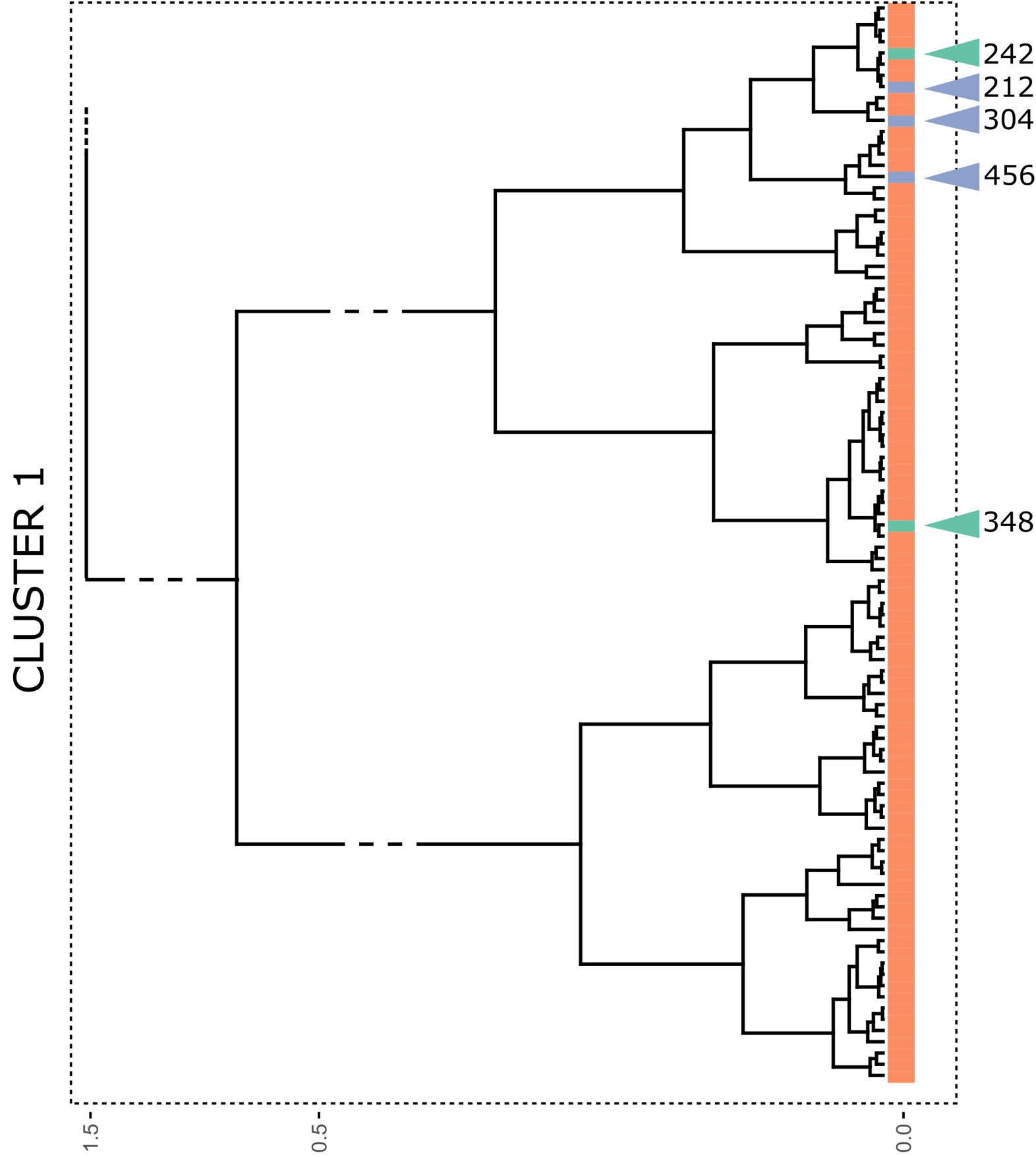

Supplement: Supplementary file 7 — Supplementary Material 7. a and b. Hierarchical dendrogram showing the similarity-based relationships between all included subjects. Three clusters were automatically created without a priori by separating the deepest branches in the tree to optimize the relative loss of inertia. Colors display the original diagnosis proposed by the experts (green: controls, blue: metopic ridge, orange: trigonocephaly). When diagnosis did not match the cluster prediction, the patient was marked with a triangle, and age in days was indicated. [file 13023_2024_3197_MOESM7_ESM.pdf]

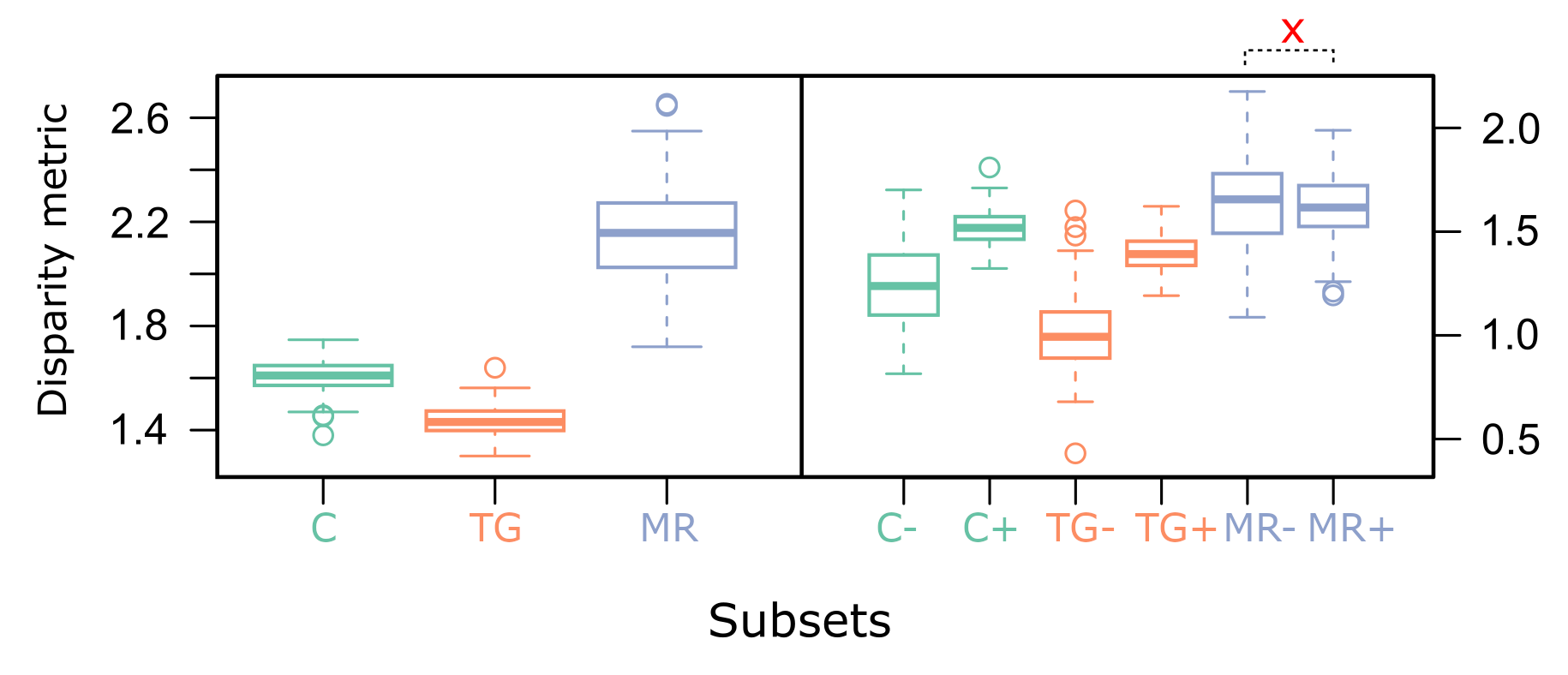

Supplement: Supplementary file 8 — Supplementary Material 8. Comparison of morphological disparity metrics between diagnosis groups (MR, metopic ridge; C, control; TG, trigonocephaly) (on the left), and depending on age (+: after 10 months of age; -: before 10 months of age) (on the right). All subsets were significantly different (all p<0.05) from each other, except between MR+ and MR- (red cross). [file 13023_2024_3197_MOESM8_ESM.png]
