# Supplementary material for: New diagnostic criteria for metopic ridges and trigonocephaly: a 3D geometric approach
Source: Orphanet J Rare Dis. 2024 May 18;19:204. doi: 10.1186/s13023-024-03197-8 (PMC11102612; doi:10.1186/s13023-024-03197-8)
Supplement: Supplementary file 9 — Supplementary Material 9. Dependency of prediction accuracy to age (<10 months vs > 10 months). Younger patients were most often misattributed than patients >10 months of age, even though the overall classification skills of the algorithms were satisfactory (MR, metopic ridge; C, control; TG, trigonocephaly). [file 13023_2024_3197_MOESM9_ESM.docx]

|  | Training set: complete  Testing set: complete | | | Training set: complete  Testing set: <10 months | | | Training set: <10 months  Testing set: <10 months | | |
| --- | --- | --- | --- | --- | --- | --- | --- | --- | --- |
|  | Accuracy 100% | | | Accuracy 93% | | | Accuracy 87% | | |
|  | MR | C | TG | MR | N | TG | MR | N | TG |
| MR | 4 | 0 | 0 | 0 | 0 | 0 | 0 | 0 | 0 |
| C | 0 | 19 | 0 | 2 | 21 | 0 | 4 | 20 | 0 |
| TG | 0 | 0 | 16 | 1 | 0 | 20 | 1 | 1 | 19 |
